# Applications of Human Skin Microbiota in the Cutaneous Disorders for Ecology-Based Therapy

**DOI:** 10.3389/fcimb.2020.570261

**Published:** 2020-10-22

**Authors:** Hong Zhou, Linlin Shi, Yuanyuan Ren, Xi Tan, Wei Liu, Zhi Liu

**Affiliations:** ^1^College of Life Science and Technology, Huazhong University of Science and Technology, Wuhan, China; ^2^National Engineering Research Center for Nanomedicine, Huazhong University of Science and Technology, Wuhan, China

**Keywords:** human skin microbiota, cutaneous disorders, dysbiosis, ecology-based therapy, applications

## Abstract

The skin represents the exterior interface between the human body with the environment while providing a home to trillions of the commensal microorganisms—collectively referred to as the skin microbiota. These microbes that coexist in an established balance play a pivotal role in the protection of cutaneous health and the orchestration of skin homeostasis. However, the well-controlled but delicate balance can be perturbed by alterations in the skin microbial communities, namely, dysbiosis, often due to commensals defeated by pathogens competing for space and nutrients, which leads to the occurrence of multiple cutaneous disorders. In view of this, the analysis of skin microbiota constituents in skin diseases is crucial for defining the role of commensal microbes and treatment of skin diseases. Emerging evidence shows that the ecology-based therapy of microbial transplantation has been proven as a valid therapeutic strategy for cutaneous disorders caused by skin microbial dysbiosis. Although its mechanism is not well-understood, there are already some applications for ecology-based therapy with the aim of correcting the imbalances on the cutaneous ecosystem. In this review, we summarize the interactions between dysbiosis and the cutaneous disorders, including homeostasis and dysbiosis of skin microbiota, microbial composition in skin diseases, and the mechanisms and applications of reversing or ameliorating the dysbiosis by the targeted manipulation of the skin microbiota, which may contribute to aid development of therapeutic modality for ecology-based therapy.

## Introduction

**Figure 1:**
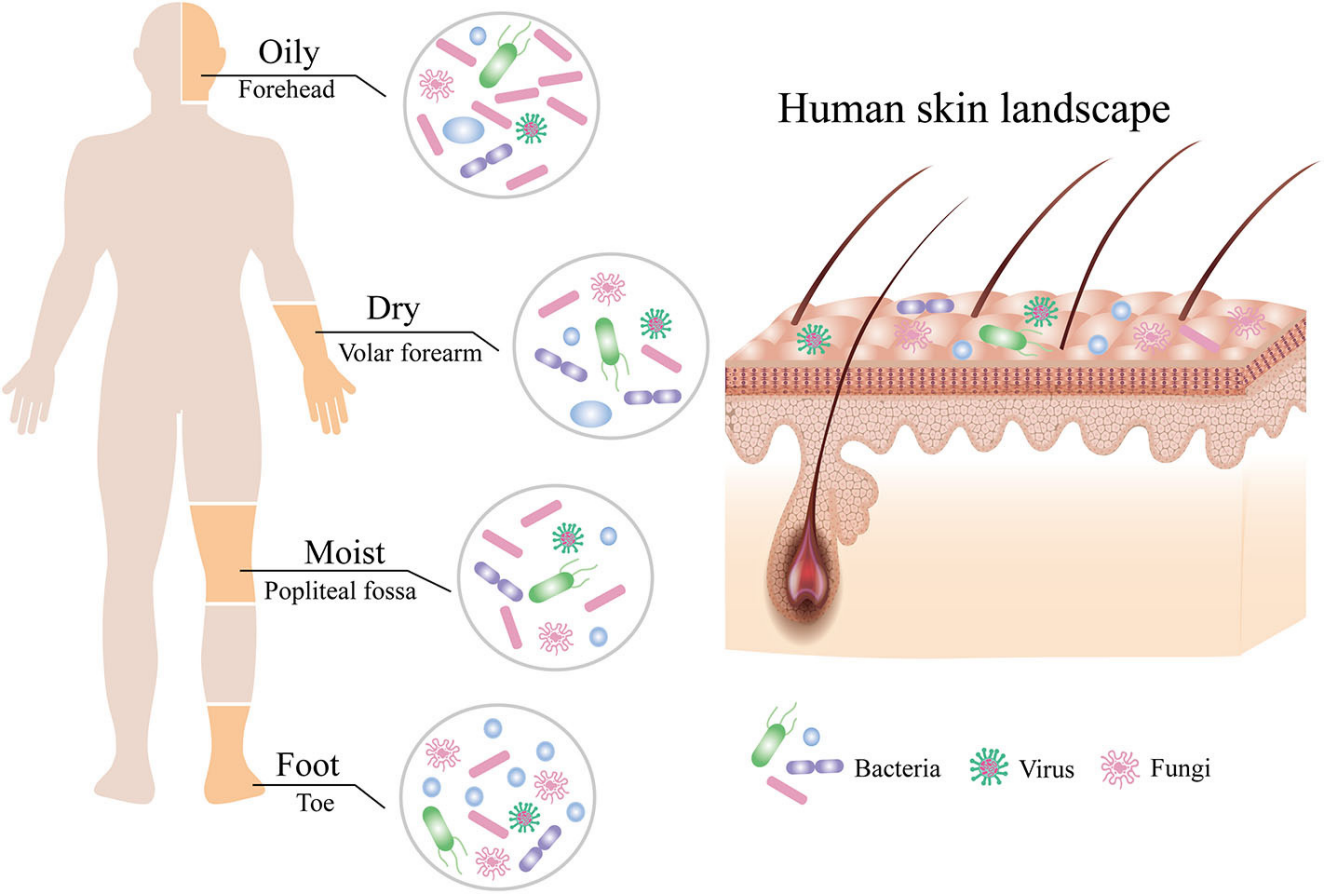
Composition of the human skin microbiota. The human skin is inhabited by millions of bacteria, fungi, and viruses that compose the skin microbiota. The composition of human skin microbiota is shaped by biogeography of the skin site including oily, moist, dry, and foot sites. Bacteria are the most abundant at all sites, followed by fungi, with the fewest viruses.

The skin is a dynamic and steady ecology that is populated with millions of microbes, such as bacteria, fungi, and viruses, which were collectively termed the skin microbiota (Figure 1; Grice and Segre, 2011). Although the skin, the largest and most exposed organ in the body (Gallo, 2017), acquires plenty of transient species of microbe by constant dialogue of different people and the external environments (Oh et al., 2016), the composition of the skin microbiota remains surprisingly stable over time (Dorrestein et al., 2016; Lax et al., 2017; Sohn, 2018). However, the diversity and relative abundance of the body's microbial communities vary in both the individual and the physiology of the skin sites, which has been categorized into four major skin microenvironment: oily, moist, dry, and foot (Figure 1; Belkaid and Segre, 2014; Byrd et al., 2018). Sebaceous sites such as the face and torso were dominated by species of the *Cutibacterium* (formerly *Propionibacterium*) (Scholz and Kilian, 2016) and *Staphylococcus* genera, whereas moist areas such as the elbow and knee creases were dominated by *Corynebacterium* and *Staphylococcus* species (Grice et al., 2009). In contrast to bacterial communities, fungi of the genus *Malassezia* are found throughout the body (Oh et al., 2014) but predominated at oily sites such as the face and back (Findley et al., 2013). Although microbial variations of these sites are highly consistent between people, the relative abundance of the individual skin microbiota, especially low-abundance microbial species, can be differentiated by metagenomic shotgun sequencing of high-resolution and multikingdom analyses (Oh et al., 2016), which hints that the individual may be identified from any part of the skin (Lax et al., 2017). For instance, although the taxa of bacteria that colonize the skin of normal individuals is generally similar, the skin microbiota of individuals with primary immunodeficiency displays more ecological permissiveness with altered population structures (Oh et al., 2013; Lehman, 2014).

Actually, it is still unknown how the homeostasis is maintained and shaped by the skin microbiota, but the balance between members of skin microbial communities plays a pivotal role in guarding against cutaneous disorders (Dreno et al., 2016). The opportunistic pathogen *Staphylococcus aureus* asymptomatically colonizes more than 30% of the individuals under steady-state conditions in a relatively low colonization rate and barely detectable abundance levels (Totté et al., 2016; Totte et al., 2016), indicating that potentially pathogenic bacteria that colonize the skin of healthy individuals are influenced by the native microbial community. However, its expansion in certain contexts is able to counteract promptly the equilibrium state (Parlet et al., 2019), ultimately resulting in several skin disorders such as atopic dermatitis (AD) (Kobayashi et al., 2015) and systemic lupus erythematosus (Conti et al., 2016). Furthermore, *Staphylococcus epidermidis*, the most prominent of the coagulase-negative *Staphylococcus* (CoNS) species (Sohn, 2018), which is generally beneficial to the host, appears unexpectedly in AD flares and predominates in less severe flares (Byrd et al., 2017), implying that a disease could arise from dysbiosis of the microbial community without an invading pathogen prevailing in the community. Of note, heterogeneous *S. epidermidis* strain communities in less severe AD flares are genomically similar to nosocomial strains that are a leading cause of mortality of sepsis in newborn infants (Meisel et al., 2016). In addition to bacterial dysbiosis, many reports have suggested that fungi and viruses have an impact on skin conditions by their own changes in cutaneous microbial communities, such as *Malassezia* and eczema (Darabi et al., 2009; Chng et al., 2016) and herpesviruses and chickenpox (Chen et al., 2018). Therefore, observing disease-associated dysbiosis is profound since these interactions may augment disease severity or susceptibility or facilitate transitions from opportunistic or commensal to pathogenic.

There is no doubt that the composition of the skin microbiota can shift dramatically during the disease progression (Kong et al., 2012). Although it is not yet known that those alterations are a cause or consequence of the skin disease, certain specific species or strains of microbes have been substantially linked with specific cutaneous disorders such as eczema (Chng et al., 2016), psoriasis (Quan et al., 2019), and acne vulgaris (AV) (Szegedi et al., 2019). For example, the appearance of AD has been tightly associated with dysbiosis in the skin microbiota (Kobayashi et al., 2015). In contrast to the skin microbiota of healthy adult, this distortion in patients with more severe AD flares is mainly caused by a single clade of *S. aureus* and demonstrates a strong correlation with *S. aureus* relative abundances (Byrd et al., 2017), suggesting that disease states of skin have a specific microbiota composition differing from that of healthy skin. These findings lead to a corollary that disease-associated changes in the skin microbial community can sever itself as a “pathogen” and then conjure up a potential therapeutic strategy that adjusting the skin microbiota reverts the consistency of premorbid state by a microbial transplant on the skin. Akin to the therapeutic principle appearing in the gut, manipulating microbiota to modulate dysbiosis has been successful in fecal microbiota transplantation (FMT) for the control of the antibiotic-resistant bacteria *Clostridioides difficile* (formerly *Clostridium difficile*) infection (Allegretti et al., 2019) and more recently in leveraging probiotics such as *Lactobacillus plantarum* to prevent neonatal sepsis (Panigrahi et al., 2017). With an increasing appreciation in the contribution of microbes to skin diseases, ecology-based therapy of microbial transplantation that exploits the preferred niche of skin microbiota has also been developed for the treatment and study of diseases. Compared to traditional antibiotic therapy of directly eradicating pathogens by brute force, the advantage of this methodology is not only to reduce the formation of drug-resistant bacteria but also to avoid destroying the indigenous microbiota of patients. Therefore, finding out the extent to which microbial composition at the site of skin disorders is deciphered is remarkably important for ecology-based therapy.

## Microbial Composition in Skin Diseases

The development of high-throughput microbial genomic sequencing technique, including amplicon sequencing and whole genome sequencing (shotgun metagenomic sequencing), makes it possible to thoroughly characterize skin microbiota constituents (Byrd et al., 2018; Grogan et al., 2019). Traditionally, due to the selectivity of artificial growth conditions, capitalizing on culture-based approaches to explore skin microbiota components can underestimate the total community diversity (Kong and Segre, 2012). Hence, to bypass the biases and selection caused by strain isolation and culture (Goodman et al., 2011), next-generation sequencing techniques are prescribed to capture the complete diversity of the skin microbiome (Neuman and Koren, 2016). Amplicon sequencing is the most commonly used for characterizing skin microbiota communities by the 16S ribosomal RNA (rRNA) gene for bacteria (Jo et al., 2016) or the internal transcribed spacer 1 (ITS1) region of the eukaryotic ribosomal gene for fungi (Schoch et al., 2012). It is able to give a classification at the relative abundance of the genus and, when possible, the species level. On the other hand, shotgun metagenomic sequencing provides much higher resolution to differentiate species-, strain- and even single-nucleotide variant (SNV)-level diversification, as all genetic material in the sample is simultaneously sequenced (Oh et al., 2014, 2016; Grogan et al., 2019). Considering that most amplicon sequencing approaches are insufficient to recapitulate skin microbiome community composition at the species level, especially members of the *Staphylococcus* genus (Meisel et al., 2016), and that the important functional differences existing among strains within a species are not being resolved (Bosi et al., 2016) due to modest gene gain or loss events or even differences in gene expression among strains (Chen et al., 2018), the ability of species- and strain-level analysis of the skin microbiome population structures is crucial for defining the role of commensals and explaining the pathogenesis of skin diseases.

### Skin Microbiota Constituents in AD

AD (also known as eczema) is a paradigmatic chronic inflammatory cutaneous disease that affects 10–30% of children in industrialized countries and has a lifetime prevalence of up to a fifth of the population in the developed world (Weidinger and Novak, 2016; Tsakok et al., 2018; Guttman-Yassky et al., 2019). The skin condition is clinically characterized by intense pruritus, relapsing eczematous lesions, and a fluctuating course (Eyerich et al., 2015; Geoghegan et al., 2018), whose manifestations not only have a substantial effect on quality of life by sleep deprivation and profoundly diminished self-esteem (Simpson et al., 2016; Weidinger et al., 2018) but also increase the risk of infection and other atopic disorders (Thaçi et al., 2016). Recent work provided evidence that patients with AD have an increased likelihood of developing asthma, allergic rhinitis, and chronic sinusitis with nasal polyposis, which were previously regarded as a symbol of overall immune-system dysfunction (Thaçi et al., 2016; Sohn, 2018). The complexity of AD onset is highlighted by the heterogeneity of course, clinical severity, and treatment responses (Byrd et al., 2017; Sun et al., 2019) and has closely evolved with multiple contributing factors, including impairment of epidermal barrier function, the immunity response of T-helper 2 cell-mediated lymphocyte skewing and immunoglobulin E (IgE)-mediated sensitization, neuroinflammation involved in itch, and dysbiosis of the skin microbiota (Weidinger et al., 2018). The potential genetic determinants of AD predisposition have been identified in relation to variants of more than 30 host gene loci, including the gene encoding *filaggrin* (*FLG*, a key component of terminal differentiation and skin barrier function) (Palmer et al., 2006) and genes linked to the immune system (Paternoster et al., 2015).

**Figure 2:**
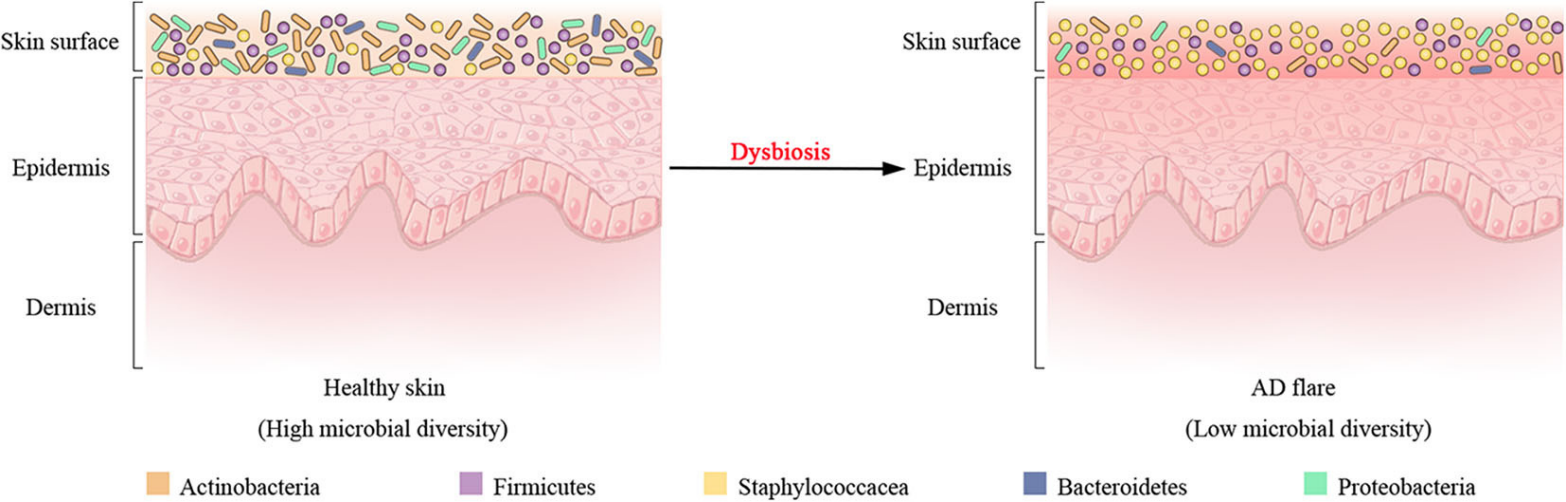
Shift of bacterial communities during atopic dermatitis (AD) disease progression. Four most prominent bacterial taxa (Actinobacteria, Firmicutes, Bacteroidetes, and Proteobacteria) and the Staphylococcaceae (a Firmicute) are found on the surface of healthy skin. Dysbiosis in AD flares is characterized by a decline in bacterial diversity and the dramatic augment in relative abundance of Staphylococcaceae.

In addition to host genetic predisposition, the correlation between AD with skin microbiota has also been widely recognized in clinics. As seen in healthy individuals, bacteria communities are the most abundant kingdom across time points and body sites for AD patients, whereas fungal communities are the least abundant and mainly *Malassezia* species, particularly *Malassezia restricta* and *Malassezia globose* (Findley et al., 2013; Chng et al., 2016; Oh et al., 2016). Human polyomaviruses and papillomaviruses prevailing in the eukaryotic DNA viral communities depend on the individual rather than anatomical site (Byrd et al., 2017). In longitudinal surveys of a cohort of pediatric AD patients, shotgun metagenomic sequencing of clinical skin sample throughout the disease course revealed that bacterial communities shifted markedly in this cohort, although the fungal or viral components had no significant differences over time (Byrd et al., 2017). For bacterial communities, investigation of AD flares demonstrated a decline in the total bacterial diversity and the dramatic increase in relative abundance of specific staphylococcal species *S. aureus* in the flare vs. the healthy (or post-flare) state (Figure 2). In addition, the relative abundance of staphylococci evolved closely with AD flare severity (Salava and Lauerma, 2014; Drago et al., 2016; Gonzalez et al., 2016). For example, individuals with less severe AD flares had relative abundances of 3.8 ± 1.7% *S. aureus* with 13 ± 3.9% *S. epidermidis*; individuals with strong AD flares had relative abundances of 34 ± 8.7% *S. aureus* with 7.4 ± 4.2% *S. epidermidis* averaged across all sites (Byrd et al., 2017). As such, these flares in eczema patients are thought to be driven by *S. aureus* skin colonization and exacerbating eczema flare-ups are associated with an increase in the number of *S. aureus* on affected region and a decrease in *S. epidermidis* that produce *S. aureus*-targeting bacteriocins. Intriguingly, a prior study had demonstrated that an increased abundance of *S. aureus* preceded flares in AD patients (Kennedy et al., 2017), hinting that *S. aureus* may contribute to the initiation of the disease rather than the consequence of the outbreak. At the strain level, a single clade of *S. aureus* primarily colonizes more severe AD patients during disease flares, and these same strains at the postflare still persisted in 80% severe AD patients but at markedly lower mean relative abundances (Byrd et al., 2017). The results of strain tracking were well confirmed using a complementary approach in which SNVs were identified in the 1.9 Mbp core genome shared between all sequenced *S. aureus*. In contrast to clonal *S. aureus* strain communities, AD patients' heterogeneous *S. epidermidis* strain communities in both flares and post-flares were composed of multiple different strains from diverse clades (Byrd et al., 2017). Strikingly, in addition to strain-specific differences during the course of AD, whole genome sequencing profiling of strain-level gene variation showed that individuals with less severe AD were colonized with more methicillin-resistant strains, whereas individuals with more severe AD were predominantly colonized with methicillin-sensitive strains (Hsiang et al., 2012; Chaptini et al., 2016).

However, recent studies hint clues that fungi might also play a fundamental role in the protection of skin from AD. *Malassezia* is a typically dominant skin-dwelling fungal class comprising 17 species and linked with AD (Sohn, 2018). One cue is that *Malassezia* peaks in greasy skin in infants or adolescence, but AD becomes less common (Havlickova et al., 2008; Seebacher et al., 2008). *Malassezia* leverages the lipids of sebum and the stratum corneum to generate their own lipids in the carbohydrate-deficient and lipid-rich skin setting (Scharschmidt and Fischbach, 2013), which concurs with an enrichment of lipase genes and a depletion of carbohydrate-utilizing enzyme genes in *Malassezia* spp. genomes (Wu et al., 2015). This indicates that the decrease in surface lipids may restrict *Malassezia*'s ecological competitiveness. Another clue found a decreased relative abundance of *Malassezia* as the population of *S. aureus* rise in AD flares (Chng et al., 2016). Noteworthy, *Malassezia* species (such as *Malassezia dermatitis* and *Malassezia sympodialis*) were enriched in AD-prone skin by whole metagenome profiling (Casagrande et al., 2006; Chng et al., 2016), suggesting that the relative abundance of *Malassezia* species shifts in association with the AD disease course. Additionally, a recent study shows that *Malassezia globosa* can secrete protease that attenuates *S. aureus* biofilm associated with increased severity and barrier dysfunction of the progression of AD (Li et al., 2018).

Additional clinical studies showed that the composition of the skin microbiota is different in pediatric vs. adult AD (Shi et al., 2016), suggesting that the disease prevalence of AD may have a linkage with age. AD most often develops in early childhood, and up to 70% of AD children demonstrate clearing of the disease or a spontaneous resolution until late childhood even in patients with *FLG* mutations (Margolis et al., 2014; Mortz et al., 2015; Bieber et al., 2017). AD can persist into adulthood in certain AD children or begin in adulthood (Garmhausen et al., 2013). The most recent estimate of prevalence of AD in the adult population is ~10% (Weidinger et al., 2018). In terms of the overall diversity of the skin microbiota, AD is significantly lower in adults than in children. Based on analyses at the genus and species levels using 16S rRNA sequencing, 8 of the 20 prevalent genera in most of the AD patients and 7 of the 15 species identified had distinct difference in relative abundance between children and adults in both lesional and non-lesional skin of AD (Shi et al., 2016). In a separate study, whole metagenome sequencing analysis that compared the non-flare skin of AD adults with that of a control cohort revealed skin microbiome-dependent susceptibility in AD flares and identified a distinct AD-associated skin microbiota signature, which had an enrichment of *Streptococcus* and *Gemella* reported to be originally enriched in children and a depletion of *Dermacoccus* often present on healthy human skin (Oh et al., 2012; Chng et al., 2016; Kennedy et al., 2017).

### Skin Microbiota Constituents in AV

AV (also commonly called acne) is one of the most ubiquitary non-communicable inflammatory dermatoses of the pilosebaceous unit (Williams et al., 2012; Lichtenberger et al., 2017), characterized by both specific localization on skin regions of abundant sebaceous glands (such as the face, neck, and back) and manifestations within a narrow age range in association with adolescence followed by frequent resolution (Szegedi et al., 2019). AV affects up to 85% of adolescents of varying ethnicities and persist into adulthood with a prevalence in 11% (Cunliffe and Gould, 1979; James, 2005; Jahns et al., 2012). The severity of AV clinical symptoms depends on the number of non-inflammatory lesions (closed and open comedones), inflammatory lesions (pustules and papules), and the residual pathology of nodules and cysts (Shalita, 2004; Ghodsi et al., 2009). Severe manifestations of AV not only have an impact on patients' physical and psychological health including abscess, permanent scarring, and depression (Zaenglein et al., 2016) but also produce social handicaps such as higher rates of unemployment (Lichtenberger et al., 2017). Although the etiology and pathogenesis of AV are not well-understood, multifaceted involvements encompassing androgen-induced increased sebum secretion and sebaceous gland proliferation, hypercornification of the pilosebaceous follicle, inflammation involving innate and acquired immunity response, skin microbiota colonization and proliferation in the pilosebaceous unit, and external factors and genetics are considered as the mechanisms contributing to the development of AV (Agak et al., 2018; O'Neill and Gallo, 2018; Chien et al., 2019).

However, with the further development of sequencing technology and the current understanding of AV pathogenesis continuously evolving, skin microbiota are considered to play a major role in the occurrence of acne. Several notable observations that the composition of the skin microbiota in acne is disturbed highlight the importance of studying diseases in the context of the microbial dysbiosis (Lomholt and Kilian, 2010; Fitz-Gibbon et al., 2013). In particular, *Cutibacterium acnes*, the most abundant commensal (up to 90%) in the skin-residing microorganisms of healthy adults, has been viewed as an important pathogenic factor accompanying the development of AV (Melnik, 2015; Barnard et al., 2016; Lomholt et al., 2017). In a case–control study utilizing immunofluorescent labeling to visualize different *C. acnes* phylotypes in macrocolonies/biofilms in sebaceous follicles of facial skin biopsies from AV patients, AV development was unequivocally linked with the occurrence and localization of *C. acnes* in follicles, and the formation of *C. acnes* biofilms attached to the hair shaft and follicular epithelial wall increased morbidity of AV patients (Jahns et al., 2012). A recent comprehensive review proposes a new concept that AV may be a naturally developing, transient inflammatory interplay of adolescent facial skin with its new microbiota (*C*. *acnes*), replacing a state of previous skin homeostasis in childhood (Szegedi et al., 2019). It is worth noting that initial attempts failed to consolidate the relationship between AV and the skin microflora. Almost all adults are colonized with *C. acnes*, but only a minority suffer from acne. Comparing the makeup of the skin microbial communities between control and AV-prone groups by next-generation sequencing technology, the results demonstrated the relative abundance of *C. acnes* tending to be consistent in both, which also implied that the skin microbiota might not play a role in AV after all (Sohn, 2018).

**Figure 3:**
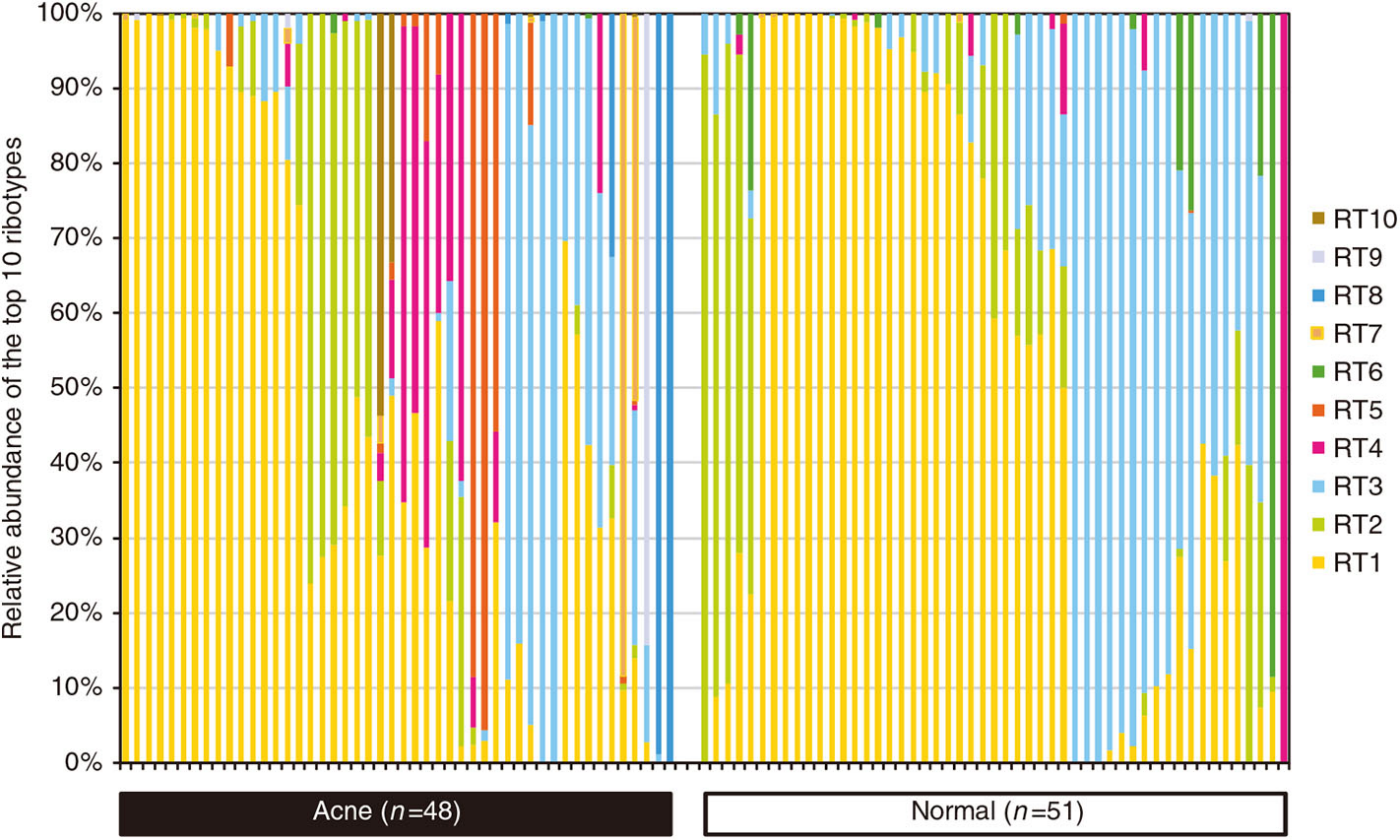
Percentage of relative abundance of diverse *C. acnes* strains in the pilosebaceous units between acne patients and normal individuals. *C. acnes* predominates the microbiota of pilosebaceous units, accounting for up to 90%. There was no significant difference in the relative abundance of *C. acnes* between acne patients and normal individuals. However, the distributions of distinct *C. acnes* strain populations in acne and normal skin are dramatically different. Dysbiosis in acne vulgaris (AV) is characterized by a decrease in the proportion of *C. acnes* strains RT6 and the significant increase in the proportion of *C. acnes* strains RT4, RT5, RT7, RT8, RT9, and RT10. Reprinted with permission from Fitz-Gibbon et al. (2013). Copyright (2013) The Society for Investigative Dermatology, Inc.

In contrast to the interspecies dysbiosis of skin microbiota in AD, the distortion of AV is caused by the shifts of intraspecies population structures of skin bacterium *C. acnes* (Fitz-Gibbon et al., 2013). Comparing the population structure of *C. acnes* strains in pilosebaceous units from 48 acne patients and 51 normal individuals using a combination of metagenomics and genome sequencing, metagenomic data analysis demonstrated that the strain-level distribution of *C. acnes* was different enough to distinguish acne skin from healthy skin (Figure 3; Fitz-Gibbon et al., 2013). Moreover, the severity of acne caused by *C. acnes* is typically strain specific (Allhorn et al., 2016). According to the top 10 most abundant ribotypes (RT) showing healthy and acne-specific associations, the three most abundant ribotypes (RT1, RT2, and RT3) had a similar relative abundance and evenly distributed in acne and normal pilosebaceous units (Fitz-Gibbon et al., 2013; O'Neill and Gallo, 2018). However, RT4 and RT5 were significantly enriched in up to 40% of AV individuals but rarely found in normal individuals. In contrast, RT6 was found to be enriched in 99% of individuals with healthy skin. At the clade level, RT4 and RT5 are categorized as the type IA-2 phylogroup that have been consistently associated with acne by increasing inflammation based on the presence of putative virulence factors (Fitz-Gibbon et al., 2013; O'Neill and Gallo, 2018). At genome and function level, the potential genetic elements and gene expression profiles of healthy and acne-associated *C. acnes* strains were revealed. The unique genome regions of acne-enriched RT4 and RT5 strains are implicated in a linear plasmid (loci 3) encoding a *tight adhesion* (*Tad*) locus in relation to virulence affecting bacterial adhesion and host immune responses and two unique loci of genomic islands (loci 1 and 2) encoding a *Sag* gene cluster in association with hemolytic activity in pathogens (Fuller et al., 2002; Humar et al., 2002; Tomich et al., 2007; Fitz-Gibbon et al., 2013; Kasimatis et al., 2013). All the genomes of healthy-enriched RT6 strain can encode *Clustered Regularly Interspaced Short Palindromic Repeats* (*CRISPRs*), which are present in health-associated type II *C. acnes* phenotypes, confer protective immunity against the invasion of viruses, phages, and plasmids or other exotic DNA, and avoid the acquisition of extra genetic elements that foster virulence and acne pathogenesis (Bolotin et al., 2005; Brouns et al., 2008; Bruggemann et al., 2012; O'Neill and Gallo, 2018). These are consistent with the results obtained from the strain-level distribution of *C. acnes*.

## Applications for Ecology-Based Therapy

**Figure 4:**
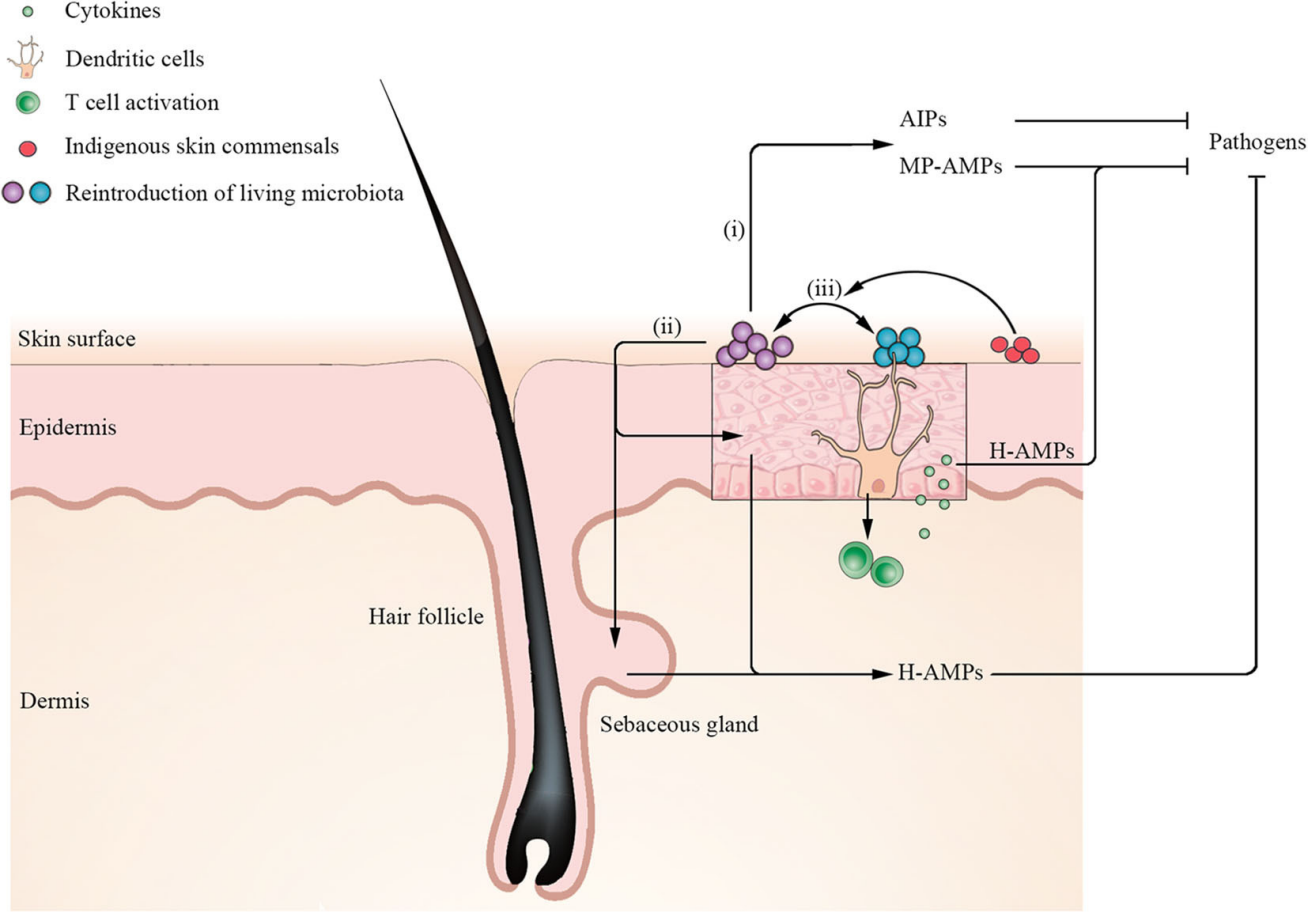
Mechanisms for establishment and restoration of cutaneous homeostasis by reintroduction of living microbiota. Application of a single of microbiota such as *S. hominis* secretes microbiota-produced antimicrobial peptides (MP-AMPs) or other metabolites [such as autoinducing peptides (AIPs)] to inhibit pathogen *S. aureus*. Moreover, reintroduction of living microbiota induces keratinocytes and sebocytes to secrete human AMPs (H-AMPs) to directly inhibit pathogens. Notably, dendritic cells (DCs) extend their dendrites into the stratum corneum to acquire microbial antigens and active T cells to release cytokines. Keratinocytes-produced H-AMPs LL-37 induced by cytokines synergize with MP-MAPs (such as *S. epidermidis* lantibiotics), which can kill *S. aureus* more effectively. In addition, reintroduction of a cocktail of microbiota has a synergistic effect on ameliorating the ecology of skin microbial communities. When certain indigenous skin commensals are rich in recipient skin, reintroduction of a cocktail of microbiota has a higher engraftment.

The understanding of the essential role of the homeostasis and dysbiosis of skin microbiota in skin disorders is rapidly expanding, with a major contributory factor being the potentiated awareness of the cutaneous ecosystem (Grice and Segre, 2011). Although there is no consistent definition of a healthy cutaneous ecosystem, some of the main parameters, such as microbial composition, diversity, and stability, have been established as key markers of homeostasis (Vandegrift et al., 2017). Disturbances of the skin microbiota have been directly correlated with multiple diseases. As a result, the novel concept of restoring the composition and functionality of the skin microbiota to the previous indigenous state by targeted manipulation of the skin microbiota, on the basis of the skin ecosystem, has been proposed as a potential therapeutic approach in the last few years (Paetzold et al., 2019; Stacy and Belkaid, 2019). As initial studies of microbiota focused on the gut, which has the largest microbial community in the human body, FMT, a synthetic reproduction of the complex gut ecosystem remaining as most effective therapeutic option with remarkable safety performance, has been applied into clinical practice although not approved by the US Food and Drug Administration (FDA) for clinical use (Cammarota et al., 2017; Allegretti et al., 2019). At present, reintroduction of living microbiota (a single or cocktail of microbiota) to modulate skin microbiota composition from disease microbiota states to healthy ones may represent a valid target for ecology-based therapy (Stacy and Belkaid, 2019). Recent advances in unlocking a key understanding of the cellular mechanisms through which the microbiota implement both the establishment and restoration of cutaneous homeostasis highlight three indispensable essential interactions (Figure 4): (i) antimicrobial peptides (AMPs) or other metabolites produced by reintroduction of a single of microbiota directly inhibit or kill pathogenic microorganisms (Nakatsuji et al., 2017, 2018; Williams et al., 2019); (ii) reintroduction of living microbiota induces keratinocytes and sebocytes to produce AMPs to shape microbial communities (Nagy et al., 2005, 2006; Naik et al., 2015); and (iii) reintroduction of a cocktail of microbiota has a synergistic effect on ameliorating the ecology of skin microbial communities (Paetzold et al., 2019). However, the extensive communication at the molecular level in the ecology of skin microbial communities is not well-understood, especially the intercellular communication of multidirectional signals. Although research on the skin microbiota lags behind studies of gut flora, there are already some applications for ecology-based therapy with the aim of correcting the imbalances on the cutaneous ecosystem.

As mentioned earlier, *S. aureus* has been implicated in the pathogenesis of AD, and the clinical severity of AD is positively correlated with carriage of *S. aureus*. Therefore, defeating the microbes in competition might contribute to treating the cutaneous disorder. Nakatsuji et al. (2017) isolated the CoNS collected from the skin of normal and AD individuals using high-throughput screening for antimicrobial activity against *S. aureus*. The predominant species of CoNS with antimicrobial activity were identified as *S. epidermidis* and *Staphylococcus hominis*, although many strains of the CoNS species did not produce antimicrobial activity. A single application of *S. hominis* A9 with anti-*S. aureus* activity confirmed a significant decrease in *S. aureus* colonizing mice and pig skin, comparing with application of *S. hominis* isolates without antimicrobial activity. Then, reintroduction of antimicrobial CoNS strains to human skin of AD subjects diminished *S. aureus* colonization. The interspecies interaction largely depends on antimicrobial CoNS strains producing novel AMPs (such as lantibiotic, bacteriocin, and lugdunin) and synergizing with microbiota-produced AMPs and the human AMP LL-37 or/and DCD-1(L) to selectively kill or inhibit *S*. *aureus* (Figure 4; Lee et al., 2008; Nakatsuji et al., 2017; Bitschar et al., 2019). Subsequently, Gallo et al. (Sohn, 2018) has devised a cream that contains antimicrobial CoNS strains isolated from human skin to inhibit *S. aureus*. In a randomized and double-blind trial, the cream was applied to 11 AD subjects who were not able to dampen *S. aureus* before accepting Gallo's intervention. The result uncovered a more than 90% decrease in the number of *S. aureus* on their skin by a single application. In follow-up studies, it was revealed that the colonization by *S. aureus* was almost completely eliminated, and clinical symptoms in AD severity declined by up to 30% when the product was applied twice a day for a week. Then, he has founded a company with the aim of bringing this product to market in the next 2–3 years. This is also basically the first time that a microbial transplantation on human skin has been useful for treatment (Sohn, 2018).

The molecular mechanism of accessory gene regulatory (agr) quorum sensing system plays a pivotal role in the orchestration of cellular behavior between CoNS species and *S. aureus* via an autoinducing peptide (AIP) signaling molecule. The full length of AIP is composed of 7–12 amino acids with the last 5 amino acids forming a cyclic (thio)lactone ring between the C-terminal and a cysteine or serine side chain (Janek et al., 2016; Zipperer et al., 2016; Nakatsuji et al., 2017). Prior studies from Dr Richard Novick's group have established the central role of agr quorum sensing signaling in *S. aureus* skin pathogenesis by several notable observations (Parlet et al., 2019), implying that the quorum sensing interaction may lead to disease or promote remissions. Otto et al. (2001) and Olson et al. (2014) applied the CoNS strains *S. epidermidis* agr type I to successfully suppress *S. aureus* agr function. Recently, Paharik et al. (2017) found that the CoNS species *Staphylococcus caprae* isolated from goat milk secreted an AIP (YSTCSYYF) that could block the agr-mediated quorum sensing of all classes of *S. aureus* (type I–IV) to prohibit their colonization and skin infection. Although a number of animal CoNS species have been identified to have inhibitory effects on *S. aureus* quorum sensing, there are still few studies on quorum sensing between CoNS species on human skin. In a more recent study, Williams et al. (2019) applied the CoNS strains collected from human subjects to identify the molecular mechanism for the effects of *S. aureus* on the epidermal barrier disruption in the development of AD and further determine how dysbiosis on the skin surface permits the bacteria to induce inflammation. Under the control of the agr quorum sensing system, the human CoNS species produced AIP to suppress the expression of *S. aureus* PSMα to prevent *S. aureus*-mediated epithelial damage and inflammation. In addition, the topical application of live CoNS strain *S. hominis* C5 producing an AIP (SYNVCGGYF) to murine skin resulted in potent inhibition of *S. aureus* agr activity, demonstrating that the human skin microbiota can contribute to epithelial barrier homeostasis by using quorum sensing to dampen *S. aureus* virulence factors (Williams et al., 2019).

Previous studies have illustrated that dysbiosis may lead to carcinogenesis. For instance, the abnormal proliferation of *Fusobacterium nucleatum* in the gut has been linked to colorectal cancer through their adherence and invasion into intestinal epithelial cells, ultimately resulting in increased oncogenic and inflammatory responses (Song et al., 2019), which suggests that certain members of human skin microbiota may curb tumor growth. Recently, Nakatsuji et al. (2018) found that a strain of *S. epidermidis*, which is common on human skin, produces 6-N-hydroxyaminopurine (6-HAP) that has selective antiproliferative activity against tumor lines by interfering with the essential process of DNA replication but did not inhibit primary keratinocytes. A single application of *S. epidermidis* strain producing 6-HAP to mouse impaired the incidence of ultraviolet-induced skin tumors. However, further research is needed to validate whether the dysbiosis caused by the loss of such strains elevates the risk of human skin cancer.

In addition to CoNS, the potential role of another Gram-negative skin bacteria in AD is also investigated. *Roseomonas mucosa* is one particular commensal found on the skin of healthy and AD individuals (Paller et al., 2019). In mouse and cell culture models of AD, *R. mucosa* isolated from healthy individuals improved the prognosis, but those collected from AD patients worsened the prognosis, whose interventions of targeting the microbiome could provide therapeutic benefit for AD patients (Myles et al., 2018). Subsequently, Myles et al. (2018) tested the therapeutic potential of topical live *R. mucosa* in humans for the first time. In an open-label phase I/II safety and activity trial in 10 adult and 5 pediatric patients, topical microbiome transplantation with *R. mucosa* led to a prominent reduction in AD severity and *S. aureus* burden without any negative reactions or treatment complications. Besides, metabolites of *R. mucosa* on the non-lesional and lesional skin of AD patients demonstrated that differences at strain level might contribute to disease pathology. These observations mirror AD treatment effects of other skin bacteria present in healthy and AD individuals but lack known cellular and molecular mechanisms.

Human AMPs (such as cathelicidins and β-defensins) are produced by keratinocytes and sebocytes to potentiate the skin resistance against pathogens. Except for the constitutively expressed AMPs, others can be agitated by cues from certain members of the skin microbiota, a phenomenon known as heterologous protection. For example, these cues can be directly stimulated by *S. epidermidis* TLR2 signaling or induced by activation of *S. epidermidis*-specific IL-17^+^CD8^+^ T cells that confer protection against skin infection by inducing keratinocytes to secrete cathelicidin and kill distinct pathogens (Figure 4; Naik et al., 2015). Furthermore, *S. epidermidis* decreases the anionic charge of its surface and membrane by an AMP-sensing system called aps, thereby avoiding harmful effects on itself via decreased attraction or repulsion of human AMPs (Otto, 2009). In another *in vitro* study, two clinical isolates of *C. acnes* type IA and IB significantly induced the expression of β-defensin-2 in human sebocytes and keratinocytes (Nagy et al., 2005, 2006). The additional observation that distinct strains of *C. acnes* may differ in contribution to the pathogenesis of AV raises the exciting possibility that investigators need to identify *C. acnes* at the strain level with a greater potential to be implicated in the condition (Nagy et al., 2006).

In contrast to transplantation of a single microbiota, reintroduction of a cocktail of microbiota has a greater advantage in the level of engraftment. As investigators usually focus only on the most critical microbe that causes disease, dysbiosis on the surface of skin is thought to be the loss and increase in a single commensal microbe rather than a mix of different skin microbiota. Paetzold et al. (2019) prepared probiotic solutions that were mixtures of different skin microbiome components taken from a healthy donor to modulate the subpopulation of skin microbiota on sebaceous-gland-rich skin regions. After sequential applications of probiotic solutions containing different combinations from one strain to multiple strains of *C. acnes* on 18 subjects in the first 3 days, the observation demonstrated that the level of engraftment was positively correlated with the presence of several different strains in the probiotic solution, implicating that different strain combinations have synergistic effects on colonization of recipient skin surface and modulation of the *C. acnes* population at the strain level is feasible by microbiome transplantation. Notably, patients with *C. acnes* subtype H1 and *Leifsonia* being more abundant showed a markedly higher transplantation by using a multistrain donor solution, suggesting that different microbiota might have a synergistic effect (Figure 4; Paetzold et al., 2019). However, the correlation of multidirectional signals in the ecology of skin microbiota is poorly understood.

## Concluding Remarks and Future Perspectives

The examples discussed herein illustrate that determining the fate of the healthy or disease states of skin may need to be updated to include the skin microbiota constituents. The available evidence has clearly indicated that the dysbiosis of cutaneous microbiota is substantially associated with multiple skin disorders, and the degree of disorder has a positive correlation with the severity of skin disease. One limitation of the current study is that the skin microbiota in dysbiosis do not differentiate well from that in healthy states. This information would provide a supporting evidence that the differences in species- and strain-level composition for the effects on the host and other skin microbiota have been largely unexplored, only mainly focusing on common skin diseases such as AD and AV (Fitz-Gibbon et al., 2013; Nakatsuji et al., 2017). Thus, further studies are needed to establish standards for skin microbial healthy states and to conduct a broader and more accurate analysis to determine the differences in multiple levels and functions of skin microbiota between dysbiosis and healthy conditions. In addition, advanced sequencing technology makes it possible to provide an analysis of the skin microbiota composition in healthy or disease states at previously unexplored resolution even including the strain level within a species (Byrd et al., 2018). With increasing identification of highly personalized skin microbial communities (Oh et al., 2016), the presence of specific strains in patients emphasizes the importance of the individuality in the disease process and in response to diagnosis and treatment, which may provide an opportunity for precision medicine in the field of skin microbiota.

Due to the high prevalence and ability to affect physical and psychological health for human (Williams et al., 2012; Weidinger and Novak, 2016), certain common cutaneous diseases related to skin microbiota remain a frontier for research. While conventional antimicrobial therapies such as oral and topical antibiotic remain the primary therapeutic method for sufferers with the cutaneous disorders, indiscriminately targeting skin microbiota and eliminating important indigenous commensal microbes remain a major drawback (O'Neill and Gallo, 2018). Recent advances, showing that the application of natural bacteria to the human skin modulates skin microbiota composition, underscore the importance for the manipulation in the indigenous microbiota of patients and highlight an opportunity to develop an ecology-based therapeutic modality that specifically target the invading pathogens while preserving indigenous commensal bystanders in diseases affecting the skin (Parlet et al., 2019; Stacy and Belkaid, 2019). As we learn more about how commensal microbiota shape the cutaneous ecosystem, we may be able to leverage this specificity by the reintroduction of living microbiota to govern the skin homeostasis. Moreover, not all strains of one species have antimicrobial activity, and the ability of inhibiting microbiota between active strains are different (Williams et al., 2019). Understanding reintroduction of living microbiota at the strain level may be critically important for correcting dysbiosis. Furthermore, it is not clear how microbiome transplantation shapes microbial communities on diseased skin via cell-to-cell communication, but the complexity of communication from intraspecies to interkingdom dense network does play an important role in maintaining homeostasis of the cutaneous ecosystem. For instance, an AIP from a CoNS species found on human skin can suppress the growth of *S. aureus* by interspecies agr quorum sensing mechanisms to protect against epidermal injury in AD, and application of a synthetic AIP to mouse skin colonized with *S. aureus* inhibited agr activity (Williams et al., 2019). It is possible that heightened understanding of the communication network of skin microbiota in molecular mechanisms will contribute to the amelioration of dysbiosis and may provide more targeted therapies for dysbiosis.

Moving forward, it is clear that the technique of skin microbiome transplantation is only at the initial stages. A temporary modulation of the skin microbiota population at the species and strain level is feasible in patients with skin disorders without an adverse event. The application of living microbiota is largely limited by *in vitro* models (such as murine skin models) that differ immunologically and structurally from the human cutaneous milieu. As investigations in the field grows, efforts will be needed to identify the efficacy of long-term application of living microbiota onto epidermal models (Williams et al., 2019) and provide a promising *in vitro* model for human therapeutic applications (Summerfield et al., 2015; Bitschar et al., 2019).

## Author Contributions

WL and ZL conceived the structure of the review. HZ drafted the manuscript. LS, YR, and XT carried out revisions of the manuscript. All authors read and approved the final manuscript. All authors contributed to the article and approved the submitted version.

## Conflict of Interest

The authors declare that the research was conducted in the absence of any commercial or financial relationships that could be construed as a potential conflict of interest.
